# Case report: From metabolic instability to remission: a case of pheochromocytoma presenting as atypical diabetes

**DOI:** 10.3389/fendo.2025.1736477

**Published:** 2026-01-09

**Authors:** Chuqing Cao, Pingyan Xue, Xuefeng Yang, Fansu Huang, Yuting Xie, Zhiguang Zhou

**Affiliations:** 1 Department of Metabolism and Endocrinology, Key Laboratory of Diabetes Immunology (Ministry of Education), National Clinical Research Center for Endocrine and Metabolic Diseases, The Second Xiangya Hospital of Central South University, Changsha, China; 2 Department of Metabolism and Endocrinology, The Second People’s Hospital of Qujing City, Yunnan, Qujing, China; 3 Department of Nuclear Medicine, The Second Xiangya Hospital of Central South University, Changsha, China; 4 Department of Clinical Nutrition, National Clinical Research Center for Endocrine and Metabolic Diseases, The Second Xiangya Hospital of Central South University, Changsha, China

**Keywords:** adrenalectomy, atypical diabetes, pancreatic β-cell function, pheochromocytoma, secondary diabetes

## Abstract

**Introduction:**

Pheochromocytoma is a well-established cause of secondary hypertension and can lead to dysglycemia. However, its initial presentation as atypical diabetes, characterized by severe glycemic instability, is uncommon and poses a significant diagnostic challenge.

**Case presentation:**

A 49-year-old non-obese Chinese woman presented with a 15-month history of progressive polyuria and polydipsia. Initially diagnosed with type 2 diabetes, she developed significant glycemic variability with recurrent hypoglycemia and profound hyperglycemia, alongside a rapid decline in β-cell function. During hospitalization, paroxysmal hypertension was observed. Biochemical testing confirmed markedly elevated catecholamines and metabolites. Imaging identified a left adrenal mass, confirmed as pheochromocytoma following laparoscopic adrenalectomy. Postoperatively, all antidiabetic medications were discontinued. At 3-month follow-up, she achieved sustained normoglycemia with restored β-cell function and normalized blood pressure.

**Conclusions:**

This case demonstrates that pheochromocytoma can induce a severe, reversible form of diabetes characterized by profound metabolic instability and marked glycemic variability. It highlights the necessity of including pheochromocytoma in the differential diagnosis of patients with atypical, labile diabetes, especially when paroxysmal symptoms are present. Early surgical intervention can reverse these metabolic derangements, often leading to diabetes remission.

## 1 Introduction

Pheochromocytoma (PCC), a catecholamine-secreting neuroendocrine tumor, is a well-established cause of secondary hypertension. Its classic manifestations include paroxysmal hypertension, headache, palpitations, and diaphoresis. Dysglycemia is also a frequent yet underrecognized complication, arising from catecholamine excess which impairs glucose homeostasis via suppressed insulin secretion, induced insulin resistance, and increased hepatic glucose production (1). Approximately 30–40% of patients with PCC have coexisting diabetes mellitus (2).

Although surgical resection is curative, diagnosis can be challenging, especially in patients without classic symptoms of PCC. In such atypical presentations, the accompanying diabetes is often misdiagnosed as type 1 (T1DM) or type 2 diabetes mellitus (T2DM), delaying idenfication and treatment of the underlying tumor.

We present a compelling case of a middle-aged woman initially diagnosed with T2DM, whose course was marked by severe metabolic instability, including marked glycemic variability, recurrent hypoglycemia, and a rapid decline in β-cell function—a presentation highly suggestive of atypical, labile diabetes. This ultimately led to the diagnosis of PCC. This case highlights that PCC can masquerade as brittle diabetes and underscores the critical importance of considering secondary endocrine etiologies in the evaluation of atypical and metabolically unstable diabetes.

## 2 Case report

A 49-year-old Chinese woman presented with a 15-month history of hyperglycemic symptoms (polydipsia, polyuria) and a 3-month history of episodic dizziness. Initially diagnosed with T2DM at a local hospital, she was started on sitagliptin and metformin, but subsequently developed recurrent unexplained hypoglycemia (documented blood glucose as low as 2.1 mmol/L). Laboratory evaluation during a prior hospitalization revealed a glycated hemoglobin (HbA1c) of 6.4%, elevated fasting (10.53 mmol/L) and postprandial glucose (20.11 mmol/L), with fasting and postprandial C-peptide levels of 681.86 pmol/L and 1045.96 pmol/L, respectively. A critical hypoglycemic workup during a severe hypoglycemic event (glucose 1.15 mmol/L) confirmed appropriately suppressed endogenous insulin secretion, with undetectable plasma insulin (<0.50 µIU/mL), low C-peptide (0.17 ng/mL), and negative β-hydroxybutyrate. Her regimen was later adjusted to cofrogliptin, acarbose, and metformin extended-release, yet she continued to experience marked glycemic variability with irregular hypoglycemic and hyperglycemic episodes (**Supplementary Figure S1**, **Supplementary Table S1**). In the three months prior to this admission, she reported recurrent dizziness occasionally accompanied by palpitations and diaphoresis, along with an unintentional 2 kg weight loss. Her medical history was negative for hypertension, dyslipidemia, or endocrine tumors.

On physical examination, her vital signs were as follows: blood pressure 123/74 mmHg, heart rate 85 bpm, height 158 cm, weight 47.5 kg, and body mass index 19.0 kg/m². Laboratory results, detailed in **Table 1**, showed an elevated HbA1c, high fasting and postprandial glucose levels, and notably low C-peptide levels. Islet autoantibodies (GADA, IA-2A, and ZnT8A) were negative. During hospitalization, she experienced an episode of dizziness with a blood pressure of 198/88 mmHg, heart rate 89 bpm; and a concomitant blood glucose of 7.2 mmol/L, which resolved spontaneously within minutes. Subsequent 24-hour ambulatory blood pressure monitoring revealed significant fluctuations, ranging from 92–203/63–125 mmHg (**Supplementary Figure S2**), which led to further evaluation on pheochromocytoma/paraganglioma (PPGL).

**Table 1:** Laboratory data on admission in this subject.

Peripheral blood	Value	Endocrinology markers	Value	Diabetes and lipid markers	Value
WBC	6,280/μl	ACTH	31.8 pg/ml	FPG	9.37 mmol/L
Platelet	23.2 × 10^4^μl	Cortisol	23.25 μg/dl	2hPG	15.4 mmol/L
RBC	389 × 10^4^/μl	DHEA-S	70 μg/dl	FCP	1.09 ng/ml
Hemoglobin	12.3 g/dl	17OHP	0.04 ng/ml	PCP	0.85 ng/ml
Hematocrit	37.1%	PRA	0.13 ng/ml/hr	HbA1c	6.9%
**Blood biochemistry**		Aldosterone	8.9 ng/dL	LDL-cholesterol	1.73 mmol/L
ALT	23.5 U/L	Adrenaline	158.3 pg/ml	HDL-cholesterol	1.90 mmol/L
AST	22.0 U/L	Noradrenaline	5945.5 pg/ml	Triglyceride	1.65mmol/l
Total bilirubin	0.70 mg/dl	Dopamine	1,057.4 pg/ml	**Electrolytes**	
Direct bilirubin	0.15 mg/dl	3-methoxytyramine	250.0 pg/ml	Sodium	139.9 mEq/L
Total protein	66.1 g/L	Metanephrine	88.6 pg/ml	Potassium	3.96 mEq/L
Albumin	41.5 g/L	Normetanephrine	1,051.5 pg/ml	Chloride	102.6 mEq/L
Creatinine	0.74 mg/dl	TSH	2.18 μU/ml	Calcium	8.96 mg/dl
BUN	27.6 mg/dl	FT4	0.8 ng/dl	IP	3.63 mg/dl
Uric acid	4.77 mg/dl	GH	2.12ug/L	**Urinalysis**	
CRP	0.008 mg/dl	IGF-1	39.8 ug/L	24-hour urinary vanillylmandelic acid	87 μmol/day
NT-pro BNP	57.2 pg/ml	IGFBP-3	2.87 mg/L	**Other factors**	
		PTH	66.5 pg/ml	NSE	6.42 ng/ml
				Gastrin 17	64.5 pg/ml
				ProGRP	0.04 ng/ml

RBC, Red Blood Cell; WBC, White Blood Cell; AST, Aspartate Aminotransferase; ALT, Alanine Aminotransferase; BUN, Blood Urea Nitrogen; CRP, C-Reactive Protein; ACTH, Adrenocorticotropic Hormone; DHEA-S, Dehydroepiandrosterone Sulfate; 17OHP, 17-Hydroxyprogesterone; TSH, Thyroid-Stimulating Hormone; FT4, Free Thyroxine; FPG, Fasting Plasma Glucose; 2hPG, 2-hour Postprandial Glucose; FCP, Fasting C-Peptide; PCP, Postprandial C-Peptide; GH, Growth Hormone; IGF-1, Insulin-like Growth Factor-1; IGFBP-3, Insulin-like Growth Factor-Binding Protein-3; NT-proBNP, N-Terminal pro-Brain Natriuretic Peptide; PRA, Plasma Renin Activity; LDL, Low-Density Lipoprotein; HDL, High-Density Lipoprotein; IP, Inorganic Phosphorus; PTH, Parathyroid Hormone; NSE, Neuron-Specific Enolase; ProGRP, Pro-Gastrin-Releasing Peptide.

Blood tests demonstrated markedly elevated plasma catecholamines and metabolites, mostly notably a profound elevation in norepinephrine (5945.5 pg/ml, reference range [RR]: 70–750 pg/ml). All catecholamine metabolites were retested and exceeded twice their upper reference limits (**Table 1**). In accordance with clinical guidelines (3), this biochemical profile confirmed the diagnosis of PPGL.

**Figure 1:**
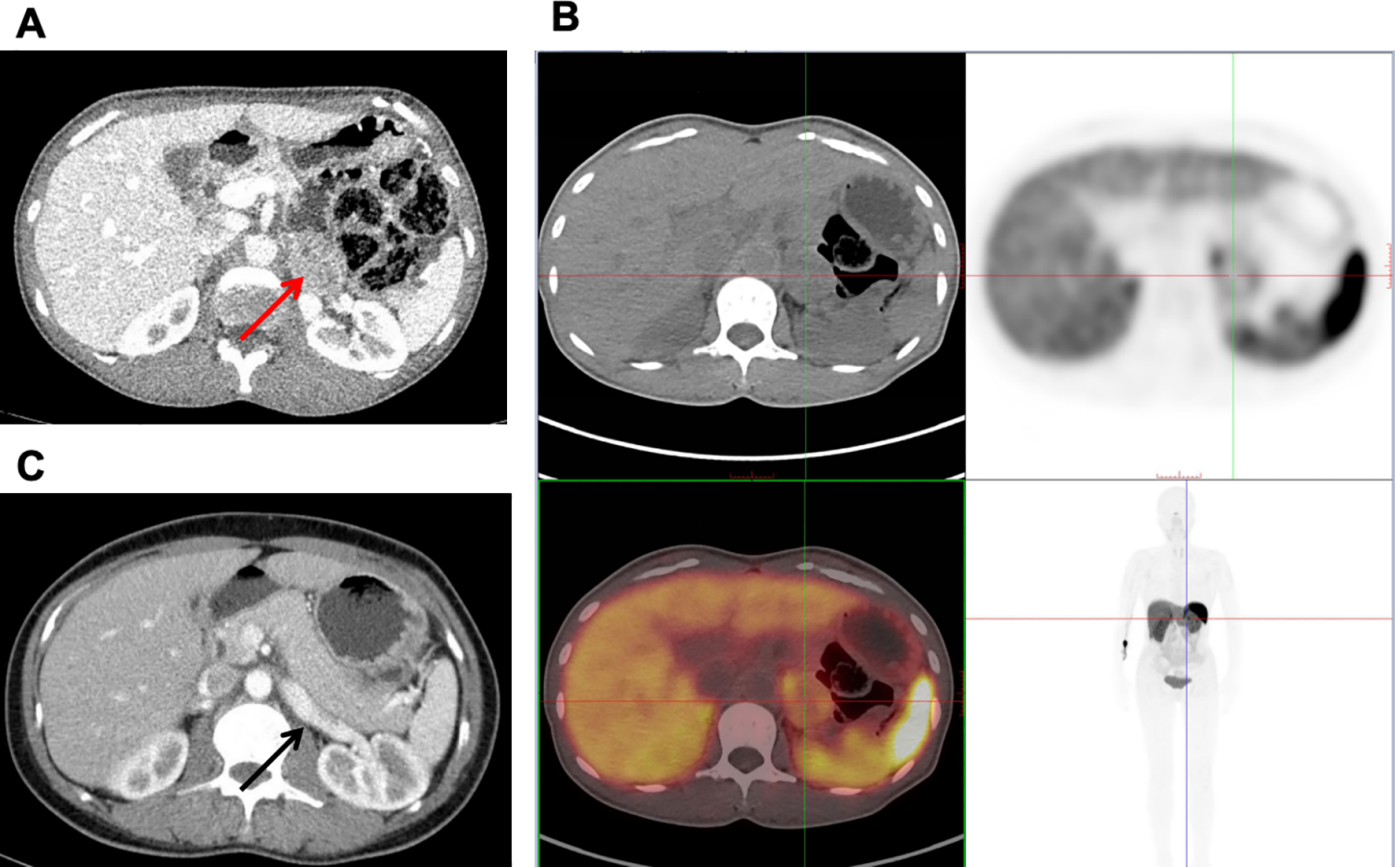
**(A)** Preoperative axial contrast-enhanced abdominal CT revealed an oval (44mm x 30mm), mildly enhancing soft-tissue lesion in the left adrenal gland (red arrow), suggestive of a pheochromocytoma. **(B)** Preoperative 68Ga-OCT PET/CT showed mild somatostatin receptor expression in the left adrenal mass, localized by crosshairs, indicative of a neuroendocrine tumor (NET). **(C)** Postoperative (3 months) axial contrast-enhanced abdominal CT demonstrated complete resection of the previous left adrenal tumor. The surgical bed shows no residual lesion or abnormal enhancement, consistent with postoperative change (black arrow).

Contrast-enhanced abdominal CT revealed a left adrenal mass (44 × 30 mm) with heterogeneous enhancement, suggestive of PCC (**Figure 1A**). To assess for potential metastasis (4), 68Ga-OCT PET/CT showed only localized somatostatin receptor expression within the mass, without evidence of metastasis (**Figure 1B**). Whole-exome sequencing identified no pathogenic PPGL-associated variants.

**Figure 2:**
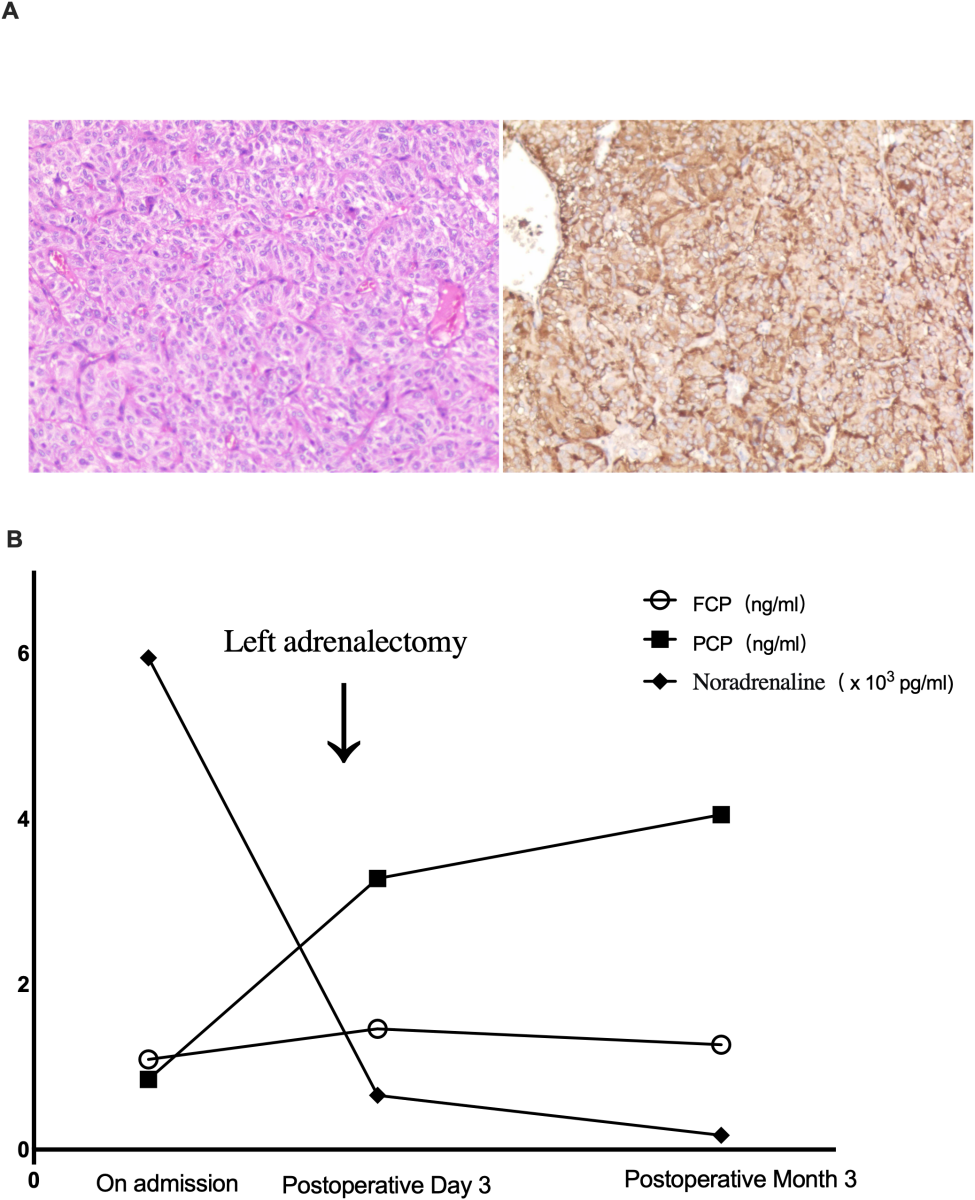
**(A)** Hematoxylin and eosin staining showed tumor cells arranged in characteristic solid alveolar nests (left panel). Immunohistochemical staining for chromogranin A (CgA) was diffusely positive, confirming the neuroendocrine origin and supporting the diagnosis of pheochromocytoma (right panel). **(B)** Time course of noradrenaline, fasting C-peptide (FCP), and 2-hour postprandial C-peptide (PCP). Noradrenaline level was drastically decreased after left adrenalectomy. Both FCP and PCP levels gradually increased after the surgery.

The patient received preoperative α- and β-blockade and subsequently underwent laparoscopic left adrenalectomy. Histopathology confirmed PCC (**Figure 2A**).

Glycemic Management and Postoperative Course: After admission, due to patient’s established glycemic pattern and her strong reluctance to initiate prandial insulin, her prior regimen of sitagliptin and acarbose were maintained initially. This was subsequently intensified to include insulin glargine and later dorzagliatin. Although provided with fixed-ratio diet, significant glucose fluctuations (from 2.7 to 17.5 mmol/L) were observed (**Supplementary Table S2**). Following tumor resection, all glucose-lowering medications were discontinued. Postoperative blood glucose levels stabilized spontaneously (3.2-11.4 mmol/L, **Supplementary Table S2**). C-peptide levels showed clear improvement by postoperative day 3 (**Figure 2B**). At the 3-month follow-up, off all antidiabetic and antihypertensive medications, she remained normal glucose metabolism, with normal HbA1c, FPG, 2hPG, and C-peptide levels on an oral glucose tolerance test (**Figure 2B**). Blood pressure remained normal, and follow-up adrenal CT showed no evidence of recurrence (**Figure 1C**), confirming PCC-induced diabetes.

## 3 Discussion

This case demonstrates that PCC can present predominantly as a form of atypical, difficult-to-control diabetes. The patient’s initial profile—absence of ketosis, preserved β-cell function, and negative autoantibodies—led to a diagnosis of T2DM at a local hospital. However, a lean body habitus, marked glycemic variability with recurrent, unexplained hypoglycemia, and a rapid decline in C-peptide levels collectively defined a non-typical, metabolically unstable diabetic phenotype. The pivotal and definitive evidence was the complete normalization of glucose metabolism following tumor resection, establishing this profound metabolic instability as a direct and reversible consequence of catecholamine excess. This outcome highlights catecholamine-induced, reversible β-cell dysfunction as the core mechanism (5) and underscores a critical clinical implication: PCC must be considered in the differential diagnosis of atypical diabetes, especially when significant metabolic instability is accompanied by episodic cardiovascular or adrenergic symptoms.

The pathogenesis of PCC-induced dysglycemia is multifactorial, driven by catecholamine excess which disrupts glucose homeostasis through coordinated pathways. The profound suppression of insulin secretion, a hallmark of this case, is primarily mediated by catecholamine action on α_2_-adrenergic receptors on pancreatic β-cells (1, 6). Catecholamines also stimulate glucagon release from pancreatic α-cells via α_1_- and β_2_-adrenergic receptors, thereby promoting hepatic glycogenolysis and gluconeogenesis (6). The resultant hormonal shift—diminished insulin with elevated glucagon—creates a potent diabetogenic state. Concurrently, catecholamines exacerbate insulin resistance by promoting lipolysis in adipose tissue (6, 7) and impairing insulin-mediated glucose uptake in skeletal muscle (6, 8) through α_1_- and β-receptors. Chronic catecholamine excess may also suppress incretin secretion, further compromising postprandial glucose regulation (9). This multi-organ disruption collectively underpins the observed metabolic instability in this patient.

A notable preoperative feature was the patient’s significant glycemic variability, including unexplained, irregular hypoglycemic episodes. The most immediate explanation is iatrogenic hypoglycemia, given the concurrent antidiabetic therapy. However, several features are less typical of purely drug-induced hypoglycemia: their severity and recurrence despite the use of agents with low hypoglycemic risk; the documented marked suppression of C−peptide during hypoglycemia; and the low fasting and stimulated C−peptide. Taken together, these characteristics also argue against mechanisms that require preserved endogenous insulin secretion, such as β_2_-adrenergic receptor-mediated stimulation or transient catecholamine negative feedback via α_2_-adrenergic receptors (1, 10). Adrenal insufficiency and growth hormone deficiency were excluded via normal cortisol and IGF-1 levels. Therefore, an alternative explanation for this patient is ectopic secretion of insulin-like growth factor 2 (IGF-2) or its precursor “big IGF-2” by PCC (11). IGF-2 exerts insulin-like bioactivity that directly induces hypoglycemia, while simultaneously suppressing endogenous insulin and C-peptide secretion—exactly matching the patient’s biochemical profile (low C-peptide and hypoglycemia) (12–14). Although IGF-2 was not assayed, this mechanism best explains the profound glycemic swings.

This patient’s favorable outcome is consistent with the literature, where surgical resection is established as the definitive treatment for PCC-induced diabetes. Large retrospective studies confirm high diabetes remission rates (78.6% to 90%) after tumor resection (15–17). The rapid recovery of C-peptide in this patient as early as the third postoperative day demonstrates how rapidly metabolic function can normalize once catecholamine excess is eliminated. This underscores a key clinical implication: timely diagnosis and surgical intervention can resolve even severe forms of PCC-induced diabetes, avoiding unnecessary lifelong antidiabetic therapy for affected patients.

This study has several limitations that should be acknowledged. Although the biochemical diagnosis of PCC was unequivocal, assays for IGF−2 were not performed, precluding a definitive assessment of their contribution to the observed glycemic dysregulation. Furthermore, without continuous glucose monitoring, we could not assess the temporal correlation between glycemic excursions and paroxysmal hypertensive episodes, a link that would have supported the proposed mechanism. In addition, the follow−up period remains relatively short. Longer observation would help confirm sustained metabolic normalization and long-term oncological outcomes.

In conclusion, this case highlights that PCC can present predominantly as an atypical, metabolically unstable diabetes. PCC should be actively considered in the differential diagnosis of any patient presenting with an atypical diabetes phenotype, marked glycemic variability unexplained by conventional therapy, a non-obese habitus, negative autoantibodies, and episodic blood−pressure surges. Recognizing this potentially curable secondary diabetes is essential for directing patients toward definitive care and improving patient outcomes.

## Data Availability

The original contributions presented in the study are included in the article/**Supplementary Material**. Further inquiries can be directed to the corresponding authors.
