# Supplementary material for: Case report: From metabolic instability to remission: a case of pheochromocytoma presenting as atypical diabetes
Source: Front Endocrinol (Lausanne). 2026 Jan 9;16:1736477. doi: 10.3389/fendo.2025.1736477 (PMC12827098; doi:10.3389/fendo.2025.1736477)
Supplement: Supplementary file 1 [file DataSheet1.docx]

***Supplementary Material***

# 1 Supplementary Figures and Tables

**1.1 Supplementary Tables**

**Table S1 Blood glucose monitoring metrics summary before this hospitalization**

| Parameters | Value | Target / Note |
| --- | --- | --- |
| **Hypoglycemic Events** | 12 episodes | – |
| **Average Event Duration** | 55 minutes | – |
| **Mean Glucose** | 8.5 mmol/L | Goal: < 8.5 mmol/L |
| **Time Above Range (TAR)** | 25% | Goal: < 25% |
| **Time In Range (TIR)** | 72% | Goal: > 70% |
| **Time Below Range (TBR)** | 3% | Goal: < 4% |
| **Tight Time In Range (TITR)** | 49% | Range: 3.9 - 7.8 mmol/L |
| **Coefficient of Variation (CV)** | 40% | Goal: < 33% |
| **Glucose Management Indicator (GMI)** | 7% | Goal: ≤ 7.0% |

**Table S2** The Patient's Point-of-Care Blood Glucose Monitoring During Hospitalization

| Date | Antidiabetic Regimen | Fasting | 2h Post-Breakfast | Pre-Lunch | 2h Post-Lunch | Pre-Dinner | 2h Post-Dinner | Pre-Bedtime | 2 AM |
| --- | --- | --- | --- | --- | --- | --- | --- | --- | --- |
| 3-19 | Acarbose 100 mg Tid, Sitagliptin/Metformin (1 tab) Bid | – | 12.2 | – | 11.2 | 13.8 | 8.2 | – | – |
| 3-20 | Acarbose 100 mg Tid, Sitagliptin 100 mg Qd, Metformin 0.5g Bid | 7.8 | 13.7 | 3.9 | 2.9 | 5.2 | 12.1 | – | 6.6 |
| 3-21 | Acarbose 100 mg Tid, Sitagliptin 100 mg Qd, Metformin 0.5g Bid | 10.4 | 15.4 | 6.1 | 6.4 | 8.3 | 10.6 | 9.2 | 7.1 |
| 3-22 | Acarbose 100 mg Tid, Sitagliptin 100 mg Qd, Metformin 0.5g Bid | 5.9 | 3.4 | 9.4 | 8.7 | 7.2 | 10.5 | 5.1 | 5.9 |
| 3-23 | Acarbose 100 mg Tid, Sitagliptin 100 mg Qd, Metformin 0.5g Bid, Insulin Glargine 2 U Qn | 7.6 | 13.3 | 10.2 | 11.4 | 8.0 | 11.4 | – | 7.0 |
| 3-24 | Acarbose 100 mg Tid, Sitagliptin 100 mg Qd, Metformin 0.5g Bid, Insulin Glargine 2 U Qn | 8.8 | 8.7 | 11.7 | 3.0 | 6.7 | 13.0 | – | 8.0 |
| 3-25 | Acarbose 100 mg Tid, Sitagliptin 100 mg Qd, Metformin 0.5g Bid, Insulin Glargine 2 U Qn | 13.0 | 8.9 | 6.2 | 13.8 | 12.3 | 7.3 | – | 3.9 |
| 3-26 | Acarbose 100 mg Tid, Sitagliptin 100 mg Qd, Metformin 0.5g Bid, Insulin Glargine 2 U Qn | 9.1 | 15.5 | 11.4 | 7.0 | 10.8 | 12.0 | – | 9.5 |
| 3-27 | Acarbose 100 mg Tid, Sitagliptin 100 mg Qd, Metformin 0.5g Bid, Insulin Glargine 2 U Qn | 10.6 | 14.6 | 10.0 | 8.9 | 7.5 | 9.7 | – | 5.7 |
| 3-28 | Acarbose 100 mg Tid, Sitagliptin 100 mg Qd, Dorziglitin 75 mg Qd, Insulin Glargine 3 U Qn | 10.7 | 17.5 | 10.6 | 5.1 | 12.2 | 9.1 | – | 7.5 |
| 3-29 | Same as above | 7.7 | 10.4 | 2.7 | 5.8 | 4.0 | 6.5 | – | 7.4 |
| 3-30 | Same as above | 7.4 | 11.1 | 9.2 | 8.9 | 7.5 | 13.8 | – | 10.1 |
| 3-31 | Same as above | 9.1 | 12.2 | 8.2 | 5.3 | – | 8.3 | – | 7.4 |
| 4-01 | Same as above | 9.2 | 7.6 | 7.6 | 7.1 | – | 6.2 | – | – |
| 4-02 | Same as above | 7.7 | 7.6 | 8.6 | 6.8 | – | 8.8 | – | – |
| 4-03 | Same as above | 8.4 | 12.4 | – | 5.3 | – | 7.8 | – | – |
| 4-04 | Same as above | 9.4 | 8.3 | 8.6 | 8.7 | – | 9.1 | – | – |
| 4-05 | Acarbose 100 mg Tid, Sitagliptin 100 mg Qd, Dorziglitin 75 mg Qd, Insulin Glargine 5 U Qn | 8.4 | 13.6 | 8.6 | 7.4 | – | 6.7 | – | – |
| 4-06 | Acarbose 100 mg Tid, Sitagliptin 100 mg Qd, Dorziglitin 75 mg Qd, Insulin Glargine 7 U Qn | 9.0 | 12.3 | 8.7 | 6.8 | – | 6.2 | – | – |
| 4-07 | Acarbose 100 mg Tid, Sitagliptin 100 mg Qd, Dorziglitin 75 mg Qd, Insulin Glargine 9 U Qn | 10.4 | 10.0 | – | – | – | – | – | 7.1 |
| 4-08 | Same as above, transferred to Urology Department | 7.8 | – | – | – | – | – | – | 10.8 |
| 4-09 | No antidiabetic medication | 3.0 | 12.2 | 5.4 | – | – | – | – | 10.4 |
| 4-10 | No antidiabetic medication (fasting, post-surgery) | Q4h glucose: 7.8 - 3.2 - 5.2 - 4.4 - 11.4 | | | | | | | |

**
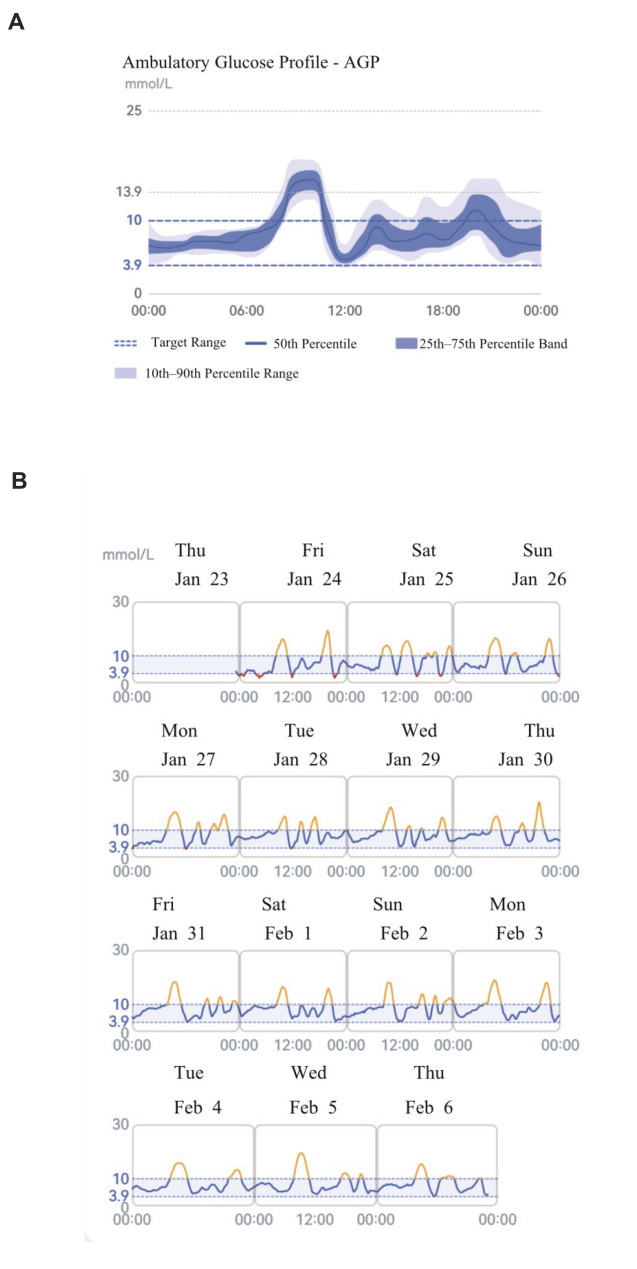
Figure S1.** Continuous glucose monitoring (CGM) data prior to hospitalization. (A) Ambulatory glucose profile (AGP). The composite 14-day AGP illustrates the overall glycemic patterns. (B) Daily glucose traces. Individual daily glucose profiles from January 23 to February 6, 2025, are displayed. Each day’s trace vividly demonstrates the extreme glycemic instability, with frequent and rapid oscillations between hypoglycemia (values at or below 3.9 mmol/L) and marked hyperglycemia (values exceeding 13.9 mmol/L).


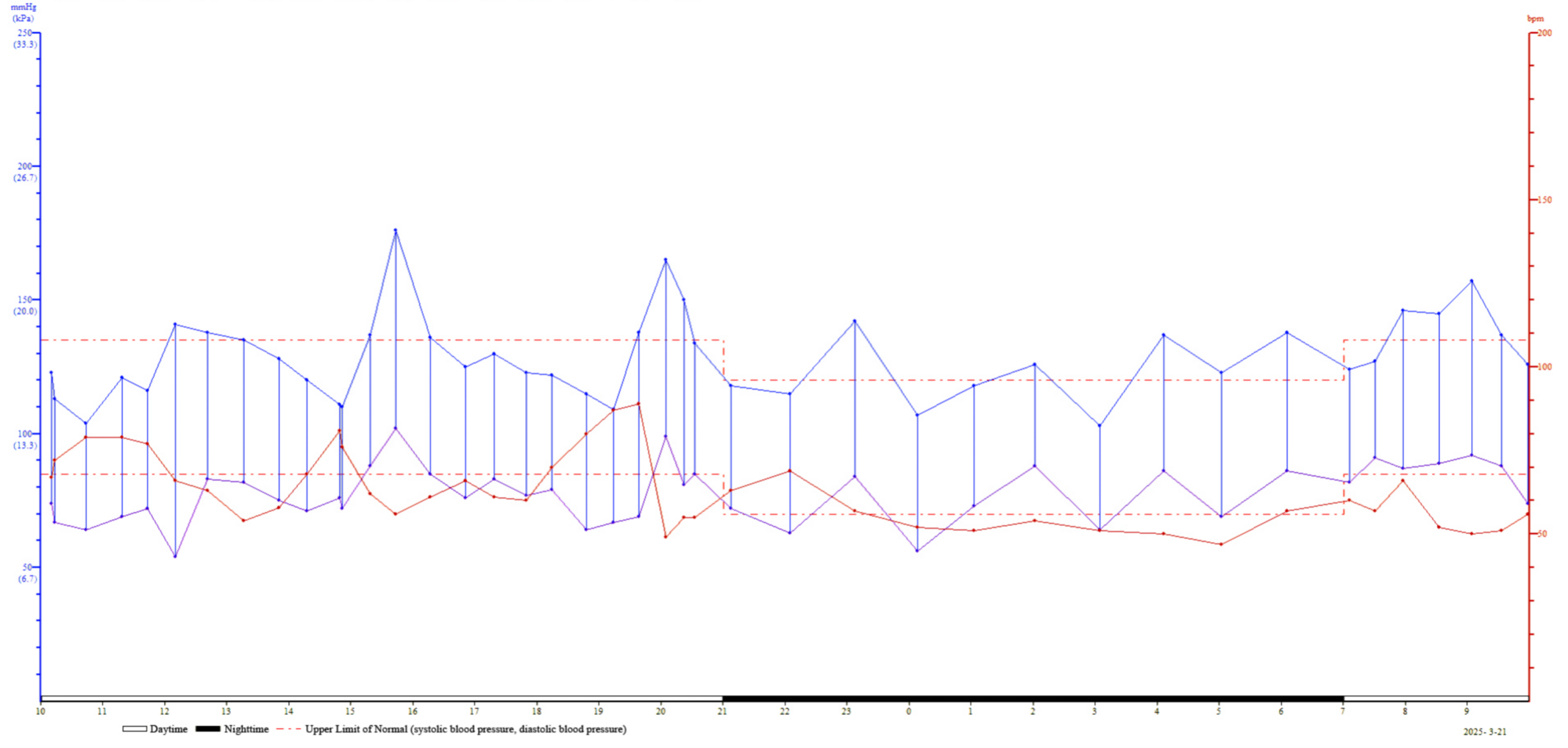


****Figure S2.** 24-hour ambulatory blood pressure monitoring. This tracing illustrates the significant blood pressure (BP) lability recorded in the patient during monitoring. The BP demonstrated marked fluctuations, with systolic/diastolic readings ranging from 92–203/63–125 mmHg throughout the 24-hour period.**
